# Clinical Significance, Differential Diagnosis and Initial Management of Hematuria in Emergency and Outpatient Settings

**DOI:** 10.7759/cureus.91639

**Published:** 2025-09-04

**Authors:** Stanislaw Szymkiewicz

**Affiliations:** 1 Department of Urology, Janusz Korczak Provincial Specialist Hospital, Slupsk, POL

**Keywords:** bladder cancer, emergency department, gross hematuria, hematuria, initial assessment, microscopic hematuria, triage, urology

## Abstract

Hematuria is a common clinical symptom that may reflect a wide spectrum of underlying conditions, ranging from benign etiologies to potentially life-threatening diseases such as urothelial carcinoma or renal trauma. It is generally classified as either gross (visible) or microscopic (detected only through urinalysis), and particularly in emergency settings, it requires prompt and structured evaluation to guide further diagnostic and therapeutic decisions. Delayed recognition may result in missed malignancies or avoidable complications, underscoring the importance of early and accurate assessment at the initial point of medical contact.

This review offers a comprehensive overview of the early management of hematuria, with particular emphasis on emergency and outpatient care settings. It highlights practical strategies for triage, differential diagnosis, and identification of high-risk patients, while providing evidence-based guidance on indications for referral and hospitalization. The analysis is grounded in a broad review of the current literature from 2016 to 2025, incorporating international guidelines and expert recommendations to ensure relevance and clinical applicability.

Ultimately, hematuria should never be underestimated during the initial clinical encounter. A systematic approach that integrates risk stratification and adherence to current guidelines allows for timely detection of serious underlying pathologies, such as malignancy or obstructive uropathy. Recognizing alarm features, such as gross hematuria, anemia, persistent symptoms, or impaired renal function, is essential for initiating appropriate diagnostic pathways, expediting specialist referral, and improving overall patient outcomes.

## Introduction and background

Hematuria, the presence of red blood cells in the urine, is a frequent clinical finding encountered in both inpatient and outpatient practice. It is broadly categorized into gross hematuria (visible to the naked eye) and microscopic hematuria (detected only through urinalysis) [1,2]. Both forms require careful evaluation, as they may reflect conditions ranging from benign and transient to serious, potentially life-threatening diseases [3].

Microscopic hematuria is observed in approximately 2-4% of the adult population during routine screening, while gross hematuria accounts for 0.2-0.3% of emergency department presentations [4,5]. Although sometimes self-limited, hematuria is clinically significant because it may be the first sign of malignancy, glomerular disease, or urolithiasis. In older adults and smokers, visible hematuria is associated with a 10-20% risk of underlying urothelial carcinoma [3,6]. Delayed recognition increases the likelihood of missed cancer diagnoses, progression of obstructive uropathy, and avoidable complications [7].

Professional societies, including the American Urological Association (AUA) and the European Association of Urology (EAU), recommend structured diagnostic pathways to improve early detection of significant pathology and reduce variability in practice [1,2,8]. Nevertheless, real-world adherence remains inconsistent, and diagnostic delays are still common [9,10]. Moreover, most available data derive from high-income settings in North America and Europe, with limited validation in resource-constrained health systems [11]. Novel diagnostic approaches, such as urinary biomarkers and artificial intelligence (AI)-assisted triage, are under active investigation but are not yet integrated into standard protocols [5,12].

This narrative review aims to provide a practical overview of the initial evaluation and management of hematuria, with particular emphasis on emergency and outpatient settings. It highlights risk stratification, diagnostic priorities, and indications for referral or hospitalization, while synthesizing evidence from recent studies and international guidelines. The focus is on strategies that can be directly applied in frontline clinical practice, rather than emerging technologies still under investigation.

## Review

Etiologies & differential diagnosis

Extrarenal and systemic causes of hematuria include coagulopathies and anticoagulant use. In addition to traditional agents such as warfarin, the increasing use of direct oral anticoagulants (DOACs), including apixaban, rivaroxaban, dabigatran, and edoxaban, has been associated with hematuria, particularly in elderly and polymorbid patients [8]. Hematuria in this context may occur even in the absence of structural abnormalities, reflecting the pharmacological effect on hemostasis rather than underlying urological pathology [9]. Nevertheless, persistent or gross hematuria in patients on DOAC therapy still warrants a complete urological work-up, as malignancy or obstructive causes can coexist. Management requires balancing anticoagulation needs with bleeding risk, often in consultation with hematology and urology. Temporary interruption or dose adjustment of DOAC therapy may be indicated in severe cases, particularly when hematuria is complicated by clot retention or anemia. Sickle cell disease or trait, particularly in individuals of African or Mediterranean descent, is a known cause due to papillary necrosis [10]. Rarely, urinary tract endometriosis may lead to cyclical hematuria, often involving bladder or ureteral implants [11]. Hematuria can also follow vigorous physical exertion and usually resolves spontaneously, though differentiation from glomerular disease is essential [12]. In pediatric populations, post-infectious glomerulonephritis, congenital anomalies, and UTIs are leading causes, with most transient cases being benign; nonetheless, serious renal disease must be excluded [12].

In summary, hematuria may be transient or persistent, symptomatic or asymptomatic, and its clinical implications depend on patient age, associated symptoms, and comorbid conditions. Early classification into glomerular, non-glomerular (urological), or systemic causes, as recommended by the American Urological Association [7] and the European Association of Urology [1], allows for appropriate diagnostic strategies and specialist referral. Table 1 summarizes these categories, their representative conditions, and distinguishing clinical features [1,5,6].

**Table 1:** Classification and Common Causes of Hematuria GN: Glomerulonephritis; UTI: Urinary tract infection; BPH: Benign prostatic hyperplasia; RCC: Renal cell carcinoma; RBCs: Red blood cells; RBC casts: Red blood cell casts; Proteinuria: Presence of protein in urine; Dysmorphic RBCs: Morphologically abnormal red blood cells, suggestive of glomerular origin

Category	Examples	Key Features
Glomerular (Renal)	IgA nephropathy, Post-infectious GN, Vasculitis, Alport syndrome	Proteinuria, dysmorphic RBCs, RBC casts, hypertension
Non-glomerular (Urological)	UTIs, Urolithiasis, BPH, Bladder cancer, RCC, trauma	Irritative symptoms, pain, visible blood, risk of malignancy
Extrarenal/Systemic	Anticoagulation, coagulopathies, sickle cell, endometriosis, exertion	Systemic signs, transient, medication-related, non-urological

Differentiating the underlying cause of hematuria is essential for guiding further diagnostics and therapeutic decisions. This process frequently begins in emergency or urgent care settings, where clinicians must quickly assess the severity and potential implications of hematuria to exclude life-threatening conditions and initiate appropriate management [13]. Timely differentiation helps prevent diagnostic delays that could lead to adverse outcomes, particularly in patients with malignancies, obstructive uropathy, or renal failure [14,15].

Hematuria may result from a broad spectrum of disorders, and establishing the correct etiology depends on a combination of clinical features, laboratory findings, and imaging. An early critical distinction is between glomerular and non-glomerular (urological) sources. Glomerular bleeding typically originates in the renal parenchyma and presents with dysmorphic erythrocytes or red blood cell casts on microscopy, often accompanied by significant proteinuria, hypertension, and lower extremity edema [5,16]. These findings are suggestive of nephritic syndromes such as IgA nephropathy, lupus nephritis, post-infectious glomerulonephritis, Alport syndrome, and ANCA-associated vasculitis [5,8,17]. The presence of systemic symptoms like arthralgia, rash, or elevated inflammatory markers further supports a renal etiology [9].

In contrast, non-glomerular bleeding arises from the urological tract and is usually associated with normal erythrocyte morphology. It may be accompanied by irritative voiding symptoms (e.g., dysuria, urgency), flank pain, or a palpable mass depending on the specific pathology [6,7]. Common non-glomerular causes include urinary tract infections, particularly in women and elderly patients, which often present with concurrent pyuria or bacteriuria [1,2]. Urolithiasis is another frequent etiology, especially in younger men and patients with known stone disease, typically presenting with acute, colicky flank pain and hematuria [8,11,18]. In older men, BPH may cause hematuria due to vascular engorgement, inflammation, or concomitant infection [2].

Genitourinary malignancies, including bladder cancer, renal cell carcinoma, and upper tract urothelial carcinoma, must always be considered, especially in patients with painless gross hematuria, history of smoking, or occupational exposure to aromatic amines [3,7,19]. In fact, gross hematuria remains the most common presenting symptom of bladder cancer, and its presence mandates thorough evaluation [15,20]. Instrumentation-related hematuria, following catheterization or endoscopic procedures, is typically self-limiting but should be recognized as a potential confounder during assessment [8,11].

Life-threatening causes such as renal trauma, ruptured abdominal aortic aneurysm, and massive hemorrhage from invasive tumors require immediate exclusion, especially in patients presenting with hypotension, tachycardia, or signs of ongoing blood loss [12,14]. Similarly, anticoagulation-associated hematuria can be severe, even in the absence of anatomical abnormalities, and necessitates correction of coagulopathy when clinically indicated [1,21].

In children, the differential diagnosis shifts significantly. Transient hematuria is often benign and self-limited, but glomerular etiologies like post-streptococcal glomerulonephritis, hypercalciuria, and congenital anomalies must be ruled out using age-appropriate protocols [17]. Pediatric patients with persistent hematuria or additional abnormal findings (e.g., hypertension, proteinuria) require nephrology referral.

Risk stratification is crucial in determining the urgency and modality of further work-up. High-risk features include painless gross hematuria, age over 40, male sex, history of tobacco use, occupational exposure to chemical carcinogens (e.g., benzidine, aniline dyes), personal or family history of urinary tract malignancy, chronic indwelling catheterization, exposure to pelvic radiation, use of anticoagulants or antiplatelets, abnormal imaging, or positive urine cytology [3,6,11,20,22]. According to updated guidelines from the AUA and EAU, these patients should undergo prompt cystoscopy and upper tract imaging, preferably with CT urography, as part of comprehensive evaluation, regardless of whether hematuria is gross or microscopic [1,4,10].

Conversely, patients without risk factors and with a single episode of transient microscopic hematuria may be monitored conservatively. Follow-up may include repeated urinalysis, renal ultrasound, and lifestyle modification if contributing factors such as dehydration or strenuous exercise are identified [12,21]. However, persistence of microscopic hematuria beyond three to six months, or its recurrence, warrants complete urological assessment.

Importantly, several validated clinical algorithms and scoring tools have been proposed to aid in the stratification process. These tools incorporate demographic and clinical variables to estimate the likelihood of malignancy and determine the need for cystoscopy or advanced imaging. Evidence supports that using structured triage and risk-based protocols improves diagnostic efficiency, prioritizes high-risk individuals, and minimizes unnecessary testing in low-risk populations [7,12].

Initial evaluation

Hematuria is a frequent and potentially concerning presentation in the emergency department (ED), requiring a structured and time-sensitive approach. The primary goals at first contact are to rule out life-threatening conditions, rapidly triage patients based on clinical severity, and guide appropriate diagnostic and therapeutic measures according to evidence-based protocols [6,7]. Early evaluation is particularly crucial, as delays in identifying serious underlying causes, such as malignancy or obstructive uropathy, can negatively impact patient outcomes and increase the likelihood of complications such as infection, clot retention, or irreversible renal damage [1,10,15].

History

A comprehensive clinical history is the foundation of initial evaluation. Essential elements include characterization of hematuria (gross or microscopic), timing in the urinary stream (initial, terminal, or total), associated symptoms such as dysuria, urgency, frequency, fever, flank or suprapubic pain, weight loss, and signs of systemic illness (e.g., malaise or fatigue) [4,6,9]. Documentation of recent trauma (including sports injuries and falls), recent urological procedures, catheterization, and menstruation is also critical to identify iatrogenic or gynecological causes of apparent hematuria [1,7]. Risk factor assessment should include use of anticoagulants or antiplatelet agents (e.g., warfarin, DOACs, aspirin), prior history of urolithiasis or urological malignancy, occupational exposure to aromatic amines or other urotoxic substances, and a history of tobacco use-all of which significantly influence the pre-test probability of serious pathology [5,8,11].

Examination

Physical examination should be targeted but thorough. Key components include assessment of vital signs for hemodynamic stability, abdominal and costovertebral angle tenderness (suggestive of renal or upper tract pathology), and evaluation for suprapubic tenderness or palpable bladder (suggestive of clot retention or obstruction) [21]. External genital examination may reveal meatal bleeding, trauma, or signs of infection, while in women, pelvic examination may be necessary to exclude vaginal or cervical bleeding mimicking hematuria [16]. In special populations such as pediatric or elderly patients, where clinical presentation may be subtle or atypical, a high index of suspicion must be maintained [13].

Laboratory Tests

Initial laboratory evaluation includes urinalysis with microscopy, which helps confirm the presence of hematuria and detect proteinuria, leukocyturia, nitrites, bacteria, or dysmorphic RBCs, which may suggest glomerular disease [1,7,11]. RBC casts are particularly indicative of renal parenchymal involvement and should prompt consideration of nephrologic consultation. A complete blood count (CBC) assesses for anemia or systemic inflammatory response, while renal function tests (serum creatinine, urea) and electrolytes provide insight into renal perfusion and function [3,4,11]. A coagulation profile (INR, PT, aPTT) is essential in patients on anticoagulant therapy or with bleeding diatheses. If infection is suspected, urine culture should be obtained before initiation of empirical antibiotics [2,6]. In patients at increased risk of urothelial carcinoma, such as those over 40 years old with a smoking history or occupational exposures, urine cytology can be valuable for detecting malignant cells and guiding further urologic work-up [5,8,16].

Imaging

Imaging is a cornerstone of the evaluation. Non-contrast computed tomography (CT) of the abdomen and pelvis is the gold standard for detecting urolithiasis, masses, and trauma-related injuries, offering high sensitivity and specificity [18]. In the context of traumatic injuries with gross hematuria, CT urography plays a complementary role. While initial non-contrast CT rapidly detects renal or pelvic fractures, hematomas, and calculi, contrast-enhanced CT with an excretory (urographic) phase is crucial for identifying collecting system disruptions, urinary extravasation, and vascular involvement [17]. Guidelines recommend CT urography in hemodynamically stable trauma patients with gross hematuria to fully assess the upper urinary tract and guide surgical or interventional management. In unstable patients, focused ultrasound or CT angiography may be prioritized, with urographic phases deferred until stabilization [17]. In patients for whom radiation should be minimized, such as children, pregnant women, or low-risk individuals, renal and bladder ultrasound is a safer alternative, although it has lower diagnostic yield [14].

Cystoscopy

In persistent hematuria with negative imaging or when lower urinary tract lesions are suspected, cystoscopy is indicated for direct visualization of the bladder and urethra [12,19]. This is particularly relevant in cases of painless gross hematuria or unexplained microscopic hematuria in older adults.

Emerging Diagnostic Modalities

Beyond standard methods such as cystoscopy and CT urography, newer-generation urine and blood tests are being investigated for earlier detection of bladder and prostate cancers. These include molecular and biomarker-based assays that may improve diagnostic accuracy and reduce unnecessary procedures [5,11]. Recent meta-analyses, including the BJUI Compass systematic review by Soputro et al. (2022), indicate that FDA-approved urinary biomarkers (e.g., CxBladder, AssureMDx, BTA, NMP22, UroVysion, ImmunoCyt) demonstrate moderate sensitivity (0.66-0.97) and specificity (0.58-0.83) in the evaluation of primary hematuria [13]. At present, such tests remain largely adjunctive and are not part of routine emergency or primary evaluation. For trainees, it is important to be aware of these developments, but clinical decision-making in acute care continues to rely on established approaches [2,6,12].

Red-Flag Features

Recognition of red flag symptoms, such as gross hematuria with clot retention, hemodynamic instability, evidence of upper tract obstruction, acute kidney injury, fever with systemic inflammatory response, or signs of disseminated infection, should prompt immediate urological consultation and consideration for hospital admission [2,10,13]. Specific scenarios, such as solitary kidney with hematuria or evidence of urinary tract obstruction on imaging, also warrant urgent intervention.

Conversely, asymptomatic microscopic hematuria (AMH) in younger patients without risk factors (e.g., non-smokers, no family history, normal imaging) may be monitored conservatively with outpatient follow-up and repeat testing [3,7,21]. The decision to defer extensive work-up should be individualized based on patient characteristics, comorbidities, and symptom persistence.

The use of structured triage algorithms and standardized diagnostic pathways, as advocated by the AUA and EAU, has been shown to improve diagnostic yield, optimize resource utilization, and reduce both under- and over-investigation [1,7,12]. Integration of these protocols into emergency workflows ensures timely identification of high-risk cases and prevents delays in specialist referral.

**Figure 1:**
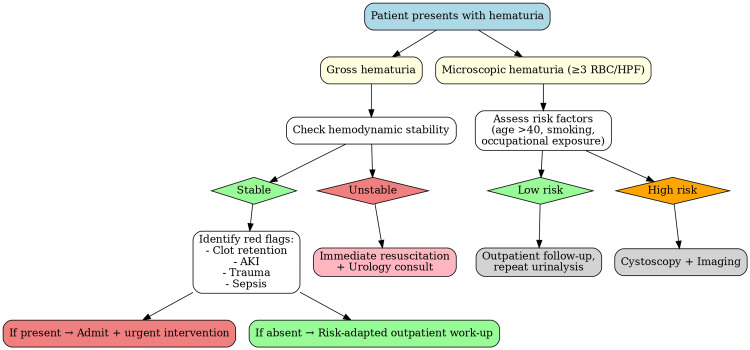
Simplified Flowchart for the Initial Evaluation of Hematuria in Emergency and Outpatient Settings RBC/HPF: Red blood cells per high-power field; AKI: Acute kidney injury

To facilitate rapid decision-making in emergency and outpatient care, a simplified clinical flowchart was added (Figure 1). This provides a stepwise overview of the initial evaluation of hematuria, highlighting key triage steps, red-flag features, and criteria for admission versus safe discharge with outpatient follow-up. Such visual tools are particularly useful for busy emergency physicians who require quick reference during patient management.

Discussion

Evidence Synthesis & Guidelines

Evidence-based clinical guidelines provide structured and stratified recommendations for the evaluation and management of hematuria, with the primary aim of early detection of significant urological pathology, particularly malignancy. Among the most influential and widely adopted are the guidelines published by the EAU and the AUA, both of which emphasize a risk-adapted diagnostic approach supported by validated algorithms [1,4,5].

According to the 2023 EAU guidelines, all patients presenting with visible (gross) hematuria should undergo a comprehensive diagnostic evaluation, regardless of age, sex, or the presence of additional symptoms. The recommended workup includes cystoscopy, which allows for direct visualization of the bladder mucosa and identification of neoplasms, inflammation, stones, or foreign bodies, and CT urography, considered the imaging modality of choice for assessing the upper urinary tract [1,19]. CT urography offers superior sensitivity and specificity compared to ultrasound, especially for urothelial carcinoma, and enables detection of subtle lesions, strictures, or calculi. When CT urography is contraindicated, such as in patients with impaired renal function, contrast allergy, or pregnancy, alternatives like renal ultrasound or magnetic resonance urography (MRU) may be employed, though with the understanding that these modalities may be less sensitive for certain lesions [4,22].

The AUA guideline, originally released in 2020 and updated in 2025 in collaboration with the Society of Urodynamics, Female Pelvic Medicine and Urogenital Reconstruction (SUFU), defines AMH as the presence of ≥3 red blood cells per high-power field (RBCs/HPF) on microscopic examination of two properly collected, non-contaminated midstream urine specimens in the absence of menstruation, infection, vigorous exercise, or other transient causes [4]. The guidelines recommend full evaluation in all adults aged ≥35 years, and in younger patients if high-risk features are present. These features include a history of tobacco use, occupational exposure to urothelial carcinogens (e.g., aromatic amines, rubber and dye industry), prior pelvic radiation, gross hematuria, or a personal or family history of genitourinary malignancy [5,11].

The recommended diagnostic workup includes cystoscopy for all patients ≥35 years or younger with high-risk features, imaging of the upper urinary tract, preferably using CT urography, though renal-bladder ultrasound may be used in low-risk or radiation-sensitive populations, and urine cytology, especially in patients at increased risk for carcinoma in situ or upper tract urothelial carcinoma [5,6].

Additionally, ancillary tests such as urine culture, renal function panels, and coagulation profiles are recommended as part of the initial evaluation to rule out infection, renal impairment, or bleeding diatheses [6,10]. In selected cases, particularly those with persistent or unexplained hematuria despite negative initial evaluations, repeat testing or advanced imaging (e.g., multiphasic CT, MR urography) may be warranted [8,12].

The review by Linder et al. (“Guideline of Guidelines”) synthesizes recommendations across major societies and emphasizes tailoring the imaging modality to the patient’s risk profile, pretest probability, and local resource availability [9]. While cystoscopy remains mandatory in most high-risk cases, ultrasound may suffice as an initial modality in low-risk patients, particularly younger females without risk factors, transient microscopic hematuria, or negative dipstick results. Some authors argue for a period of watchful waiting in these patients, with repeat urinalysis before proceeding to invasive or radiologic evaluation [21].

Both the EAU and AUA highlight the importance of timely urological referral for any patient with persistent hematuria, high-risk features, abnormal imaging, or suspicious urine cytology. Studies have demonstrated that delays in specialist referral or incomplete diagnostic evaluation are associated with more advanced stage at diagnosis and poorer oncological outcomes, especially in bladder cancer and upper tract urothelial carcinoma [16,20,23].

It should also be noted that in endemic regions, infectious causes such as schistosomiasis contribute significantly to the burden of hematuria, a factor often underrepresented in Western guidelines [24].

Importantly, the integration of clinical decision tools, such as hematuria risk calculators and flowcharts, into routine workflows can reduce diagnostic variability across care settings [7,12]. This is particularly critical in emergency departments and primary care clinics, where most patients with hematuria are first evaluated. Applying risk-based triage algorithms ensures that high-risk individuals are promptly identified and investigated, while low-risk patients are spared unnecessary testing and anxiety [10,21].

In summary, adherence to guideline-driven pathways enables clinicians to stratify risk, optimize resource utilization, and improve early detection of malignancies, thereby enhancing patient outcomes and reducing diagnostic delays. Regular updates to these guidelines, such as the 2025 AUA/SUFU revision, underscore the dynamic nature of hematuria management and the need for continued alignment of clinical practice with emerging evidence [4,5].

Hospitalization Criteria and Referral Pathways

Hospitalization should be considered in patients presenting with hematuria when clinical findings suggest serious underlying pathology or when complications arise that require immediate urological intervention [25]. The decision to admit is primarily guided by the patient’s hemodynamic status, severity of hematuria, and presence of systemic or obstructive features. Additional clinical indicators, such as significant pain, fever, vomiting, and inability to void, may also support inpatient care due to the increased risk of upper tract obstruction or systemic infection requiring parenteral treatment [6,7,14].

Patients who meet criteria for inpatient management include those with (i) hemodynamic instability (e.g., hypotension, tachycardia, signs of shock), (ii) persistent gross hematuria with clot formation and urinary retention, (iii) significant anemia requiring transfusion or evidence of active ongoing blood loss, (iv) acute kidney injury or rising serum creatinine in the context of hematuria, suggesting post-renal obstruction or nephrotoxic insult, (v) renal or pelvic trauma, particularly with gross hematuria or suspected vascular involvement, and (vi) suspected upper urinary tract malignancy with high-risk features (e.g., solitary kidney, hydronephrosis, visible mass).

In such cases, immediate urological consultation is warranted to assess the need for urgent interventions such as diagnostic cystoscopy, endoscopic clot evacuation, continuous bladder irrigation, or placement of ureteral stents or percutaneous nephrostomy tubes. In the emergency department, physicians can initiate several temporizing measures before definitive urological intervention. These include placement of a large-bore three-way Foley catheter and initiation of continuous bladder irrigation in patients with clot retention, manual irrigation for immediate relief of obstruction, administration of broad-spectrum intravenous antibiotics in cases of suspected urosepsis, and aggressive intravenous fluid resuscitation in hemodynamically unstable patients [2,6,12,14]. Indications for these interventions are primarily gross hematuria with clot retention, systemic infection with hemodynamic compromise, and acute kidney injury due to obstruction [12,14]. Clinical endpoints include restoration of urinary drainage, stabilization of vital signs, resolution of clot burden, and initiation of targeted antimicrobial therapy where infection is suspected [2,23]. These measures allow emergency physicians to bridge the critical period until definitive urological procedures can be performed. These procedures can be life-saving, particularly in patients with obstructive uropathy or severe hematuria complicated by clot retention or sepsis [2,12,14]. Studies indicate that up to 40% of patients hospitalized for gross hematuria undergo urological intervention within the first 24 hours, highlighting the necessity of timely surgical planning in ED settings [12].

In elderly or medically complex patients, even non-massive hematuria may warrant hospitalization due to an increased prevalence of genitourinary malignancies and higher risk of complications from anticoagulants, frailty, and impaired homeostasis [9,21]. Moreover, patients presenting with hematuria and signs of systemic infection, such as fever, leukocytosis, or hypotension, especially in the context of urosepsis or immunosuppression, should receive inpatient care for intravenous antibiotics and hemodynamic monitoring [1,6,8]. A frequent diagnostic grey zone arises in patients with obstructing ureteral calculi, where urinalysis may reveal hematuria with moderate leukocyte esterase but nitrite negativity. This pattern often reflects sterile pyuria caused by local inflammation from the stone rather than true infection [5,11,18]. In otherwise well-appearing and afebrile patients without leukocytosis or systemic instability, outpatient management with analgesia, hydration, and close follow-up is appropriate [6,12]. However, when such findings are accompanied by fever, rising inflammatory markers, or clinical deterioration, hospital admission for intravenous antibiotics and urgent decompression (stent or nephrostomy) is warranted [2,14,23]. Recognizing this distinction helps emergency physicians avoid unnecessary admissions while ensuring timely escalation in patients at risk of obstructive urosepsis. Hematuria in patients with indwelling catheters or post-surgical urological devices must also be assessed for potential iatrogenic injury or infection.

Conversely, patients presenting with microscopic hematuria, normal renal function, stable vital signs, and no red flag symptoms (e.g., visible hematuria, anemia, abnormal imaging) may be discharged safely from the emergency department with structured outpatient follow-up. Risk-based outpatient evaluation should include cystoscopy and upper urinary tract imaging, preferably CT urography or renal-bladder ultrasound, particularly in patients over 40 years old, smokers, or those with occupational exposure to urothelial carcinogens [3,4,5,7,8,11].

Clinical decision-making is enhanced by guideline-based risk stratification tools, which assist in identifying candidates for urgent admission versus those suitable for ambulatory management. The AUA and EAU recommend that patients with persistent microscopic hematuria and risk factors, even in the absence of ED findings, be referred for urological assessment within 2 to 4 weeks [2,3]. Algorithms incorporating age, hematuria type, symptomatology, and laboratory/imaging results improve consistency in triage decisions and reduce unnecessary hospitalizations [10,25].

Furthermore, the establishment of integrated care pathways between emergency medicine, urology, and primary care ensures that patients receive timely follow-up and appropriate diagnostic workup. Such coordination is especially crucial in the context of hematuria, where early identification of malignancy or progressive renal disease can significantly influence prognosis [1,21]. Delayed or missed follow-up, particularly in high-risk populations, has been associated with adverse outcomes, including advanced-stage urological cancers diagnosed late in the disease course [16,20,23].

Ultimately, the effective triage of hematuria requires a balance between timely intervention and avoidance of unnecessary admissions. Risk-adapted hospitalization decisions, supported by clinical guidelines and inter-specialty collaboration, are essential to improving patient outcomes and optimizing resource use in acute care settings [6,12,15,16].

Not all patients with hematuria require hospital admission; however, specific clinical indicators necessitate urgent urological intervention and inpatient care. Table 2 presents evidence-based clinical criteria for hospitalization in patients with hematuria, including hemodynamic instability, clot retention, and signs of acute renal impairment [2,6,12].

**Table 2:** Hospitalization Criteria in Hematuria

Clinical Criteria	Key Rationale
Hemodynamic instability	Shock, hypotension, tachycardia
Persistent gross hematuria with clot retention	Obstruction, need for urgent intervention
Significant anemia	Requires transfusion
Acute kidney injury	Post-renal obstruction, close monitoring
Renal or pelvic trauma	Risk of severe bleeding or organ damage
Suspected upper tract malignancy in solitary kidney	High risk of renal compromise
Intractable pain or sepsis	Systemic complications requiring inpatient care

Limitations and Future Directions

This review is based primarily on literature from high-income healthcare systems, which may not reflect diagnostic capabilities in resource-limited settings. Additionally, prospective validation of risk stratification tools across diverse populations is warranted. Future research should focus on refining biomarkers for hematuria evaluation and developing AI-assisted triage algorithms to enhance diagnostic precision.

## Conclusions

Hematuria is a common but clinically significant finding that requires timely and systematic evaluation. Visible hematuria, particularly in older adults and smokers, carries a higher malignant potential, while microscopic hematuria also warrants careful assessment. Early referral, adherence to guideline-directed pathways, and prompt recognition of red flags such as hemodynamic instability, clot retention, anemia, or renal impairment are essential for safe management. While future tools such as biomarkers and AI may enhance diagnostic accuracy, current practice should remain grounded in established methods to ensure accurate diagnosis, appropriate treatment, and improved patient outcomes.
